# Uptake and Metabolic Conversion of Exogenous Phosphatidylcholines Depending on Their Acyl Chain Structure in *Arabidopsis thaliana*

**DOI:** 10.3390/ijms25010089

**Published:** 2023-12-20

**Authors:** Ekaterina R. Kotlova, Svetlana V. Senik, Gregory A. Pozhvanov, Ilya A. Prokopiev, Ivan A. Boldyrev, Bairta S. Manzhieva, Ekaterina Ya. Amigud, Roman K. Puzanskiy, Anna A. Khakulova, Evgeny B. Serebryakov

**Affiliations:** 1 Komarov Botanical Institute, Russian Academy of Sciences, 197022 Saint-Petersburg, Russia; senik@binran.ru (S.V.S.); pozhvanov@binran.ru (G.A.P.); prokopiev@binran.ru (I.A.P.); bmanzhieva@binran.ru (B.S.M.); ekamigud@gmail.com (E.Y.A.); puzansky@binran.ru (R.K.P.); 2 Department of Botany and Ecology, Faculty of Biology, Herzen State Pedagogical University, 191186 Saint-Petersburg, Russia; 3 Frumkin Institute of Physical Chemistry and Electrochemistry, Russian Academy of Sciences, 119991 Moscow, Russia; i_boldyrev@mail.ru; 4 Chemical Analysis and Materials Research Core Facility Center, Reseach Park, Saint-Petersburg State University, 199034 Saint-Petersburg, Russia; a.khakulova@spbu.ru (A.A.K.); e.serebryakov@spbu.ru (E.B.S.)

**Keywords:** lipid uptake, plant lipids, lipidomics, phospholipids, *Arabidopsis thaliana*

## Abstract

Fungi and plants are not only capable of synthesizing the entire spectrum of lipids de novo but also possess a well-developed system that allows them to assimilate exogenous lipids. However, the role of structure in the ability of lipids to be absorbed and metabolized has not yet been characterized in detail. In the present work, targeted lipidomics of phosphatidylcholines (PCs) and phosphatidylethanolamines (PEs), in parallel with morphological phenotyping, allowed for the identification of differences in the effects of PC molecular species introduced into the growth medium, in particular, typical bacterial saturated (14:0/14:0, 16:0/16:0), monounsaturated (16:0/18:1), and typical for fungi and plants polyunsaturated (16:0/18:2, 18:2/18:2) species, on *Arabidopsis thaliana*. For comparison, the influence of an artificially synthesized (1,2-di-(3-(3-hexylcyclopentyl)-propanoate)-*sn*-glycero-3-phosphatidylcholine, which is close in structure to archaeal lipids, was studied. The phenotype deviations stimulated by exogenous lipids included changes in the length and morphology of both the roots and leaves of seedlings. According to lipidomics data, the main trends in response to exogenous lipid exposure were an increase in the proportion of endogenic 18:1/18:1 PC and 18:1_18:2 PC molecular species and a decrease in the relative content of species with C18:3, such as 18:3/18:3 PC and/or 16:0_18:3 PC, 16:1_18:3 PE. The obtained data indicate that exogenous lipid molecules affect plant morphology not only due to their physical properties, which are manifested during incorporation into the membrane, but also due to the participation of exogenous lipid molecules in the metabolism of plant cells. The results obtained open the way to the use of PCs of different structures as cellular regulators.

## 1. Introduction

As components of plasma membranes and endomembranes, glycerophospholipids (GPLs) perform many different functions in plant cells, including compartmentalization, modulation of membrane physical properties such as fluidity, flexibility, curvature, and others, targeted delivery of secretory vesicles with a specific source, and signaling where they act as first and second messengers [1,2]. Also, GPLs serve as an environment for proteins, affecting their structure and function [3,4]. These protein–lipid interactions were shown to be an important mechanism in regulating the activity of enzymes, including such enzyme superfamilies as cytochrome P450s, receptors, ion channels, and transport proteins [5,6,7]. They are involved in membrane dynamics, protein targeting, import/export machinery, and cell organelle translocation [8,9,10,11].

The molecular profiles of GPLs are highly diverse because of a combination of different blocks: two hydrophobic fatty acids, a hydrophilic polar head group attached to the glycerol backbone, and the fatty acid linkage which may be ester, ether, or vinyl–ether at the *sn*-1 position [12]. However, the latter is controversial for plants because the occurrence of ether-GPL in their cells has been reported, but not confirmed [13,14]. In plant GPLs, the *sn*-1 fatty acid tends to be saturated (16:0, 18:0), mono-(18:1), or polyunsaturated (18:2, 18:3), whereas the *sn*-2 fatty acid is more often polyunsaturated (18:2, 18:3). Heterogeneous (diacyl mixed-chain) molecular species usually contain shorter FAs at the *sn*-1 position and longer FAs at the *sn*-2 position, but in plant GPLs, the distribution of longer and shorter FAs can be reversed, e.g., as 20:X/16:X, 20:X/18:X, 22:X/18:X, 24:X/18:X [15,16]. 

**Figure 1:**
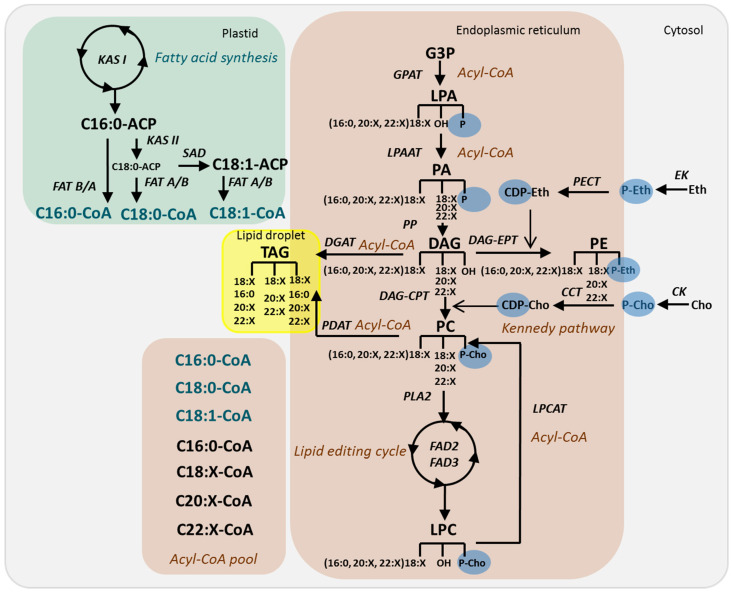
Proposed model of eukaryotic GPL synthesis and editing. Reactions in the model are designed on the basis of the literature [18,21]. Not all known reactions are represented. Acyl chains are synthetized and Δ9-desaturated on ACP backbone by means of ketoacyl-ACP synthase (KAS)I, KASII, Δ9 stearoyl-acyl carrier protein desaturase (SAD) in the plastid. Newly synthesized acyl-CoAs (green) are transported to the endoplasmic reticulum and join acyl-CoA pool (black). The reaction sequence of GPL synthesis is described in the text. Abbreviations: ACP—acyl carrier protein; CCT—CTP:phosphorylcholine cytidyltransferase; CDP-Cho—cytidine diphosphate-choline; CDP-Eth—cytidine diphosphate-ethanolamine; Cho—choline; CK—choline kinase; CoA—coenzyme A; DAG—diacylglycerol; DAG-CPT—diacylglycerol cholinephosphotrasferase; DGAT—acyl-CoA:diacylglycerol acyltransferase; EK—ethanolamine kinase; Eth—ethanolamine; AD2—oleate desaturase; FAD3—linoleate desaturase; G3P—glycerol-3-phosphate; GPAT—glycerol-3-phosphate acyltransferase; LPA—2-lysophosphatidic acid; LPAAT—2-lysophosphatidic acid acyltransferase; LPC—2-lysophosphatidylcholine; LPCAT—2-lysophosphatidylcholine acyltransferase; P-Cho—phosphocholine; P-Eth—phosphoethanolamine; PA—phosphatidic acid; PC—phosphatidilcholine; PDAT—phospholipid:diacylglycerol acyltransferase; PE—phosphatidilethanolamine; PECT—ethanolamine-phosphate cytidylyltransferase; PLA2—phospholipase A2; PP—phosphatidate phosphatase; TAG—triacylglycerol.

The structural diversity of plant lipids is primarily based on the diversity of lipid metabolism reactions. The presence of two pathways of lipid metabolism, prokaryotic and eukaryotic, also plays an essential role in lipid heterogeneity [10,17]. The prokaryotic pathway refers to the synthesis of lipids within plastids where de novo fatty acid synthesis terminating by the acyl-acyl carrier protein (acyl-ACP) occurs. Further processing of fatty acids includes the formation of phosphatidic acid (PA), phosphatidylglycerol, and glycolipids. In *Arabidopsis* leaves, approximately 40% of fatty acids synthesized in plastids, mainly 16:0 and 18:1, enter the prokaryotic pathway, whereas 60% are exported to the eukaryotic pathway [18]. In *Arabidopsis* roots or other plant species, the export of fatty acids to the eukaryotic pathway, which is localized to ER, reaches 90% or more [19,20]. Acyl-ACP thioesterases (FAT) that hydrolyze acyl-ACP determine the chain length and saturation of fatty acids: the FATA class has the highest activity for 18:1-ACP, whereas FATB prefers 16:0-ACP. The resulting free fatty acids are metabolized to form acyl-coenzyme A (acyl-CoA), which in turn is incorporated by glycerol-3-phosphate acyltransferases (GPATs) into lysophosphatidic acid (LPA). With the participation of lysophosphatidic acid acyltransferases (LPAATs), this pathway then leads to the formation of PA, which is converted to diacylglycerol (DAG) to be the precursor of the major membrane GPLs–phosphatidylcholine (PC) and phosphatidylethanolamine (PE) (Figure 1) [21].

The synthesis and remodeling of PC and PE in plants has several distinctive features. These classes of lipids are synthesized predominantly via the CDP-choline and CDP-ethanolamine pathways, respectively (together these reactions are known as the Kennedy pathway), but undergo very active and rapid remodeling. Bypass synthesis pathways, although present, do not play such an important role as in fungal and animal cells [18,22]. PCs are considered by many authors as central metabolites in donating and accepting acyl groups [21,23]. The partial hydrolysis of PC to LPC underlies many essential processes of lipid metabolism, such as the lipid editing cycle (remodeling), import and export of acyl groups between endoplasmic reticulum (ER) and plastid, and triacylglycerol (TAG) synthesis [24,25,26]. The PC acyl editing cycle includes the rapid de-acylation of PCs, the generation of LPC, accompanied by the release of fatty acids to the mixed pool of acyl-CoA, and the re-acylation of LPC by another acyl-CoA from the pool of acyl-CoA. The total rate of PC editing exceeds the level of fatty acids synthesis in developing seeds by 4 times and in developing leaves by 20 times [27].

The processes associated with the ability of plants to absorb lipids from the external environment, followed by their involvement in the metabolism, have been less studied so far. However, the presence of a well-developed root system may allow lipid absorption from the soil [21,28]. The ability of plants to assimilate exogenous lipids, including free fatty acids, GPLs, and DAG, has been demonstrated in various experiments [29,30,31,32]. The main candidate for the role of an enzyme involved in GPL transport across the plasma membrane is the flippase of the P4-type ATPase family–Aminophospholipid ATPase 10 (ALA10) [30]. Commenting on this nomenclature, it should be noted that ALA 10 can transport GPLs that do not contain amino groups such as PA and phosphatidylglycerol [30,33]. ALA 10 interrelates with the ALA-Interacting Subunit (ALIS), either ALIS1 or ALIS5, leading to differential endomembrane localizations, close to the plasma membrane with ALIS1 or to chloroplasts with ALIS5 [34]. In nongreen tissues, particularly in the epidermal cells of root tips, ALA10 naturally occurs near the plasma membrane, where it internalizes exogenous GPLs across the membrane. The uptake of phospholipids from soil humus could provide the roots with energy-rich acyl chains, phosphate groups, and some GPL derivatives, which participate in cell reception, signaling, and the metabolism. In green tissues, ALA10 is localized close to chloroplasts and may participate in MGDG biosynthesis, ensuring the translocation of PCs as a donor of acyl groups inside the chloroplast [34,35,36]. The expression of ALA10 involved in the uptake of GPLs from the external environment is tightly regulated along cell growth and differentiation and is regarded as the essential metabolic mechanism responsible for the acquisition of cell-specific phospholipid patterns.

Some of the most important sources of lipids that can potentially be utilized by plants are aquatic and soil microorganisms, particularly bacteria and fungi [37,38]. Bacterial lipids differ markedly from those of plants and fungi. The structural features of bacterial PC and PE include the presence of specific acyl groups, e.g., saturated, monoenic, cyclic, and branched [39,40,41,42]. Bacterial lipid composition is known to be highly variable because they are extremely sensitive to environmental conditions [37]. Among the factors determining microbial growth are temperature, soil–water balance, pH, carbon substrate (sugars, amino acids, organic acids), and C:N ratio [37,43,44,45]. In plant bacteria soil communities, the above factors can also affect a plant by increasing its adsorption properties and/or activating the transport systems responsible for the uptake of exogenous phospholipids [31,33,46]. In this connection, observations showing an increase in plant growth intensity (biomass, root, and leaf length) in the presence of rhizobacteria or mycorrhizal fungi are of great interest [47,48]. It is possible that, in addition to the known mechanisms enhancing plant growth through the production of indole-3-acetic acid, nitrite, etc., an important role belongs to the lipid metabolism modified by microorganisms. For example, phospholipids containing saturated, monoenoic, cyclopropane, and saturated branched acids produced by soil bacteria may have a growth-stimulating effect [48,49].

In the present paper, we tried to find out how the structure of the assimilated exogenous phospholipid affects plant growth and morphology. Previously, in a comparative study of the uptake of 16:0/06:0 NBD-PC, 06:0 NBD-lysoPC, 16:0/06:0 NBD-PE, and 16:0/6:0 NBD-PS, as well as other NBD-labeled GPLs with the same acyl groups, the preferential transport of GPLs with a choline head group was shown [30,50]. The effect of natural GPLs with different acyl groups has not been evaluated so far. In the present work, we compared the effects of exogenous PCs with acyl groups of different lengths and degrees of unsaturation, as well as cyclic PCs. Also, using different approaches, we evaluated the ability of plants to uptake and transform exogenous phospholipids. The emphasis was placed both on the ability of exogenous lipids to be incorporated into the metabolism and to induce the synthesis and remodeling responses of endogenous PCs and PEs.

## 2. Results

### 2.1. Supplementation with Exogenous PC Molecular Species Alters Seedling Macromorphology Differently

**Figure 2:**
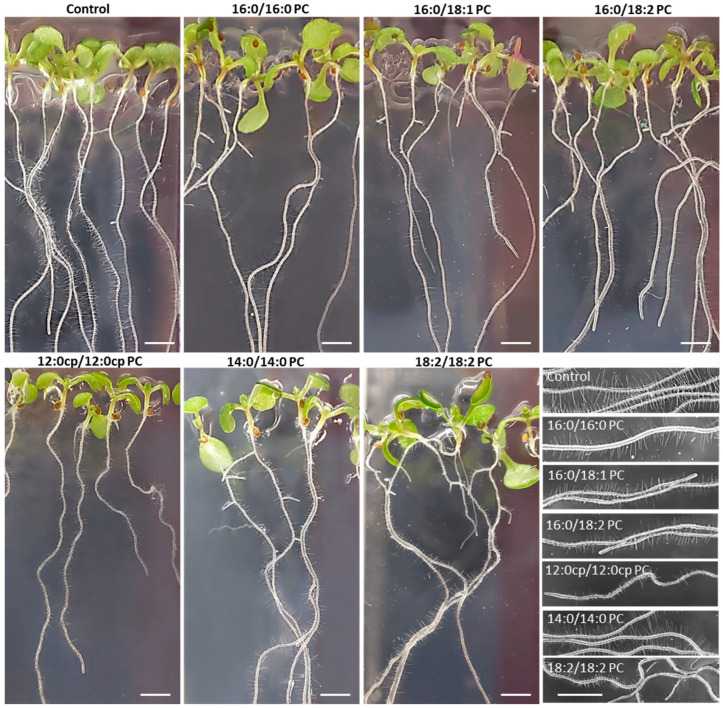
Morphology of *A. thaliana* seedlings supplemented with PC of different structures. Plants were grown in the absence (Control) and presence of 20 μM PCs at 20 °C with a 12 h/12 h light/dark cycle for 7 days (scale bar indicates 2 mm). Inset images are all of the same scale; the scale bar shown on the bottommost image is equal to 2 mm.

The exogenous PCs supplemented to the growth medium caused specific changes in seedling morphology depending on the structure of the introduced molecular species (Figure 2). In three independent experiments, the seedlings grown on the medium supplied with 16:0/18:2 PC demonstrated slower growth and shorter lengths of primary roots compared to controls. The seedlings grown on the medium with 18:2/18:2 PC had enhanced growth of the aerial parts, accompanied by maximum curvature of primary roots and rapid development of lateral ones. However, a more detailed study of 18:2/18:2 PC-exposed roots showed a change in the morphology of their root hairs, whose length was on average shorter than in controls. The root hairs of seedlings grown on the medium with 12:0cp/12:0cp PC appeared to be similar to those of 18:2/18:2 PC, but their primary roots and aerial parts were less developed. The impact of other molecular species seemed less significant, and required advanced statistical research measuring the morphological parameters of a large number of seedlings.

**Figure 3:**
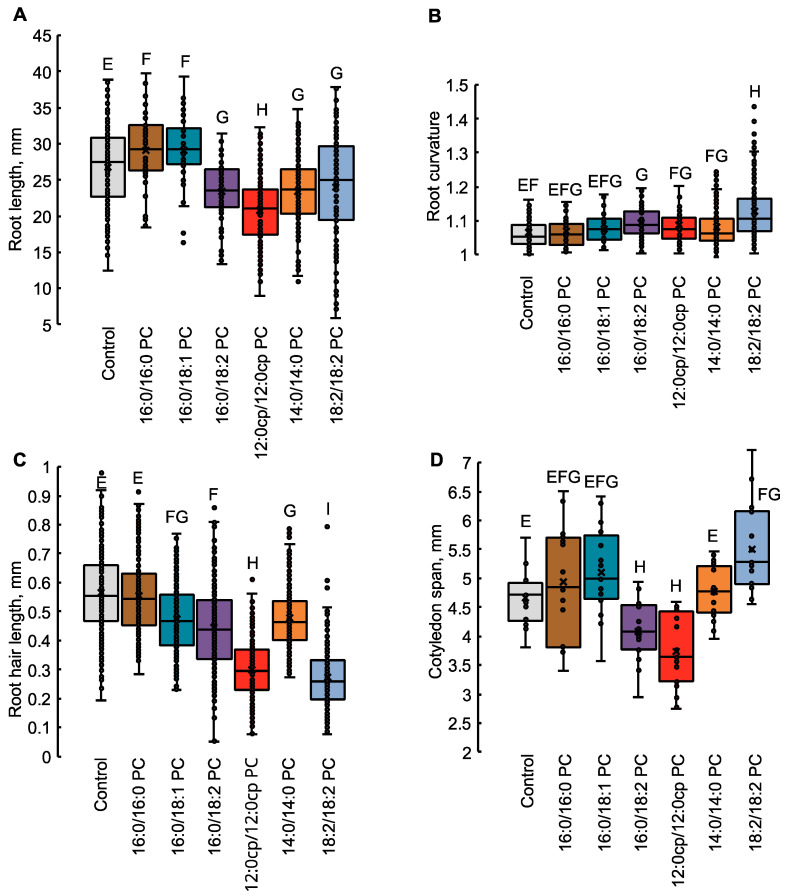
Macromorphological parameters of *A. thaliana* seedlings supplemented with PC of different structures. Data are represented as a box from 1st quartile to 3rd with a marked median. The same letters indicate values that do not differ significantly and belong to the same group; values that do not share the same letters differ significantly (one-way ANOVA, *p* < 0.05). (**A**)—root length of 7-day-old wild-type (Col-0) *Arabidopsis* seedlings grown on half-strength Murashige–Skoog medium in the absence (control) and presence of 20 μM PC at 20 °C with a 12 h/12 h light/dark cycle for 7 day; *n* = 120–188 from three independent experiments. (**B**)—root curvature of 7-day-old *Arabidopsis* seedlings; *n* = 120–188 from three independent experiments. (**C**)—length of root hairs of 7-day-old wild-type *Arabidopsis* seedlings; *n* = 232 from three independent experiments. (**D**)—cotyledon span (the distance between the two opposite cotyledon tips) of 7-day-old *Arabidopsis* seedlings; *n* = 15–21 from three independent experiments.

According to the morphometry data performed for 7-day-old *Arabidopsis thaliana* seedlings, growth on media with exogenous 16:0/18:2 PC, 12:0cp/12:0cp PC, 14:0/14:0 PC, and 18:2/18:2 PC resulted in a decrease in the length of primary roots and an increase in their curvature index, as well as a decrease in the length of root hairs (Figure 3). The seedlings grown on the media with 16:0/16:0 PC and 16:0/18:1 PC did not differ much from the control ones, except for a slight increase in the length of primary roots. Seedlings grown with 12:0cp/12:0cp PC and 16:0/18:2 PC had reduced cotyledons, whereas 18:2/18:2 PC induced a statistically significant expansion of the aerial parts. Moreover, 18:2/18:2 PC-supplemented seedlings contained a lot of lateral roots up to 1–2 mm in length. In contrast, a significant decrease in a number of lateral roots was detected in seedlings grown in 16:0/18:2 PC and 12:0cp/12:0cp PC-supplemented media.

### 2.2. Supplementation with Exogenous PC Molecular Species Alters Seedling Micromorphology

**Figure 4:**
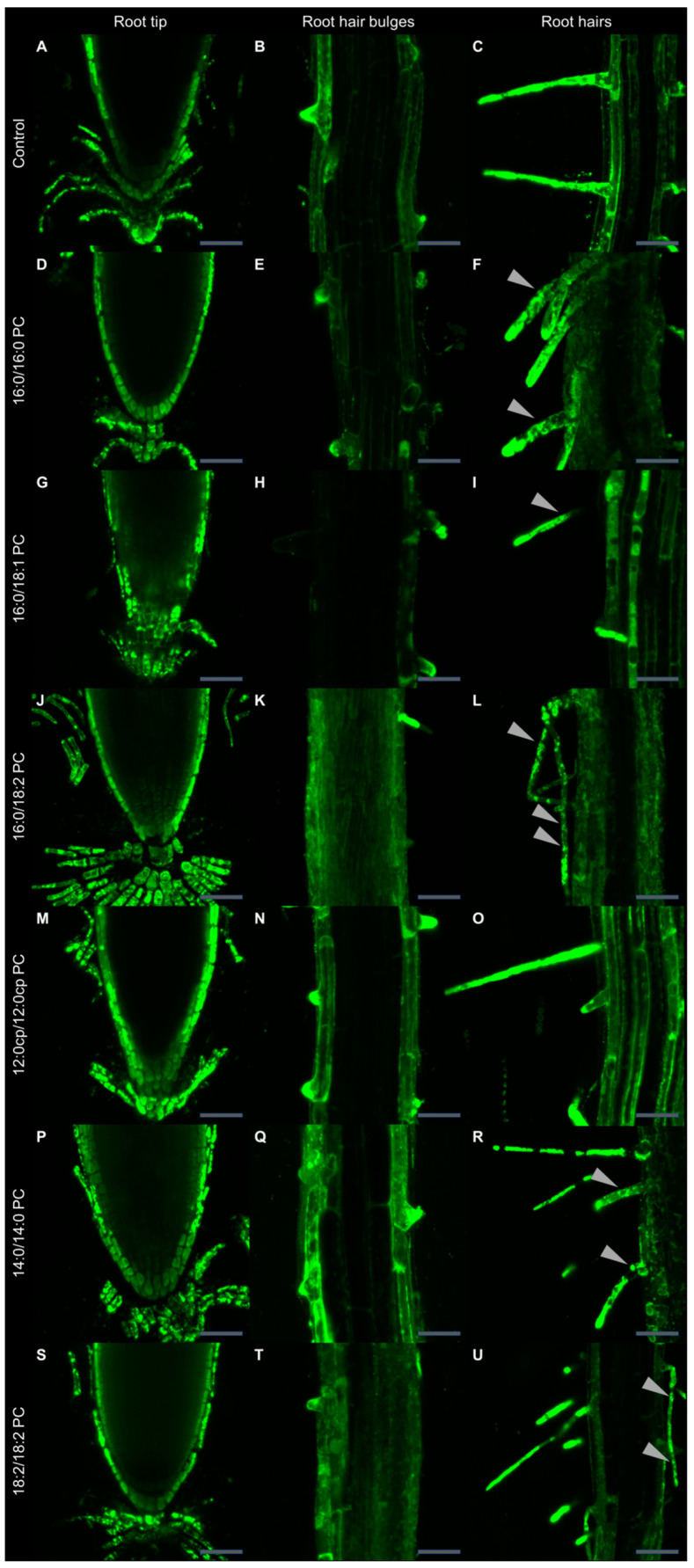
Visualization of NBD-PC uptake in *A. thaliana* roots grown on the medium supplied with various PC molecular species. Seven-day-old wild-type (Col-0) *Arabidopsis* seedlings grown on half-strength Murashige–Skoog medium in the absence (control, (**A**–**C**)) and presence of 20 μM 16:0/16:0 PC (**D**–**F**), 16:0/18:1 PC (**G**–**I**), 16:0/18:2 PC (**J**–**L**), 12:0cp/12:0cp PC (**M**–**O**), 14:0/14:0 PC (**P**–**R**), or 18:2/18:2 PC (**S**–**U**). Seedlings were bathed in 40 μM NBD-PC solution in TRIS/MES buffer (pH 6.0) for 30 min, rinsed three times, and then NBD-PC fluorescence was visualized using confocal microscopy. The leftmost column shows the root tip, the central column shows the root elongation zone with root hair bulges, and the third column depicts developed root hairs. Patches of NBD-PC staining are indicated with sharp triangles. (**K**,**L**): fluorescence signal was 160% amplified with respect to control to deliver comparable image brightness. Images represent maximal intensity projections from 4–8 optical sections. Scale bar, 50 μm.

The uptake patterns of different PCs, as well as their membrane-modulating activity, were judged by the incorporation of fluorescent NBD-PC into cells after growing plants on media with different exogenous PCs. We have inspected the fluorescence of internalized NBD-PC using confocal microscopy and found that seedlings accumulate NBD-PC mostly in physiologically active, growing parts of the root: in the root tip (apical meristem and columella), then in the elongation zone where root hair bulges emerge and develop into full-length root hairs. In control, NBD fluorescence was detected in the two outermost layers of cells (epidermis and cortex) from the root apical meristem (Figure 4A–C). Tips of root hair bulges and growing root hairs both demonstrated the highest level of fluorescence detected. Atrichoblasts and the root cortex in the elongation zone accumulated a much lesser amount of fluorescent dye. Roots grown on 12:0cp/12:0cp PC exhibited an NBD-PC staining pattern similar to that in control. In addition, NBD fluorescence in the rhizoderm of the elongation zone and in the cortical cells of the root tip was even brighter than in control roots (Figure 4M,N). Exogenous 14:0/14:0 PC resulted in the uptake of the NBD-PC similar to that in control and 12:0cp/12:0cp PC-supplemented plants -, however, some of the root hairs internalized the probe in patches (Figure 4R). The NBD-PC uptake pattern in roots grown on 18:2/18:2 PC resembled the one in control roots except for a less pronounced staining in the root hair bulges (Figure 4T). When compared to the control, roots grown on the media supplied with 16:0/16:0 PC and 16:0/18:1 PC demonstrated a shallower penetration of NBD-PC in the root tip (Figure 4D,G). NBD-PC uptake in root hairs under such treatment was focused in patches apart from the growing tip. Roots grown on 16:0/18:2 PC were characterized by the weakest and most heterogeneous inclusion of the fluorescent probe in all the active growth zones examined (Figure 4J–L). 

This study allowed the characterizing of the physical properties of root membranes in the zones of the most active absorption, including the root tip and root hairs. According to the data obtained, internalization of 12:0cp/12:0cp PC did not reduce the membrane permeability for the fluorescent lipid probe. Moreover, it can be assumed that the permeability increased in the investigated zones. Incorporation of 14:0/14:0 PC and 18:2/18:2 PC slightly decreased the membrane permeability for the fluorescent probe, but not in all areas investigated. The appearance of areas of heterogeneous staining (with patches) was registered only in the zone of root hairs. Plants growing on media with 16:0/16:0 PC and 16:0/18:1 PC altered the membrane uptake property of the fluorescent probe to a greater extent. The maximum decrease in permeability and, probably, in other physical properties of the membrane was revealed in plants supplied with 16:0/18:2 PC.

**Figure 5:**
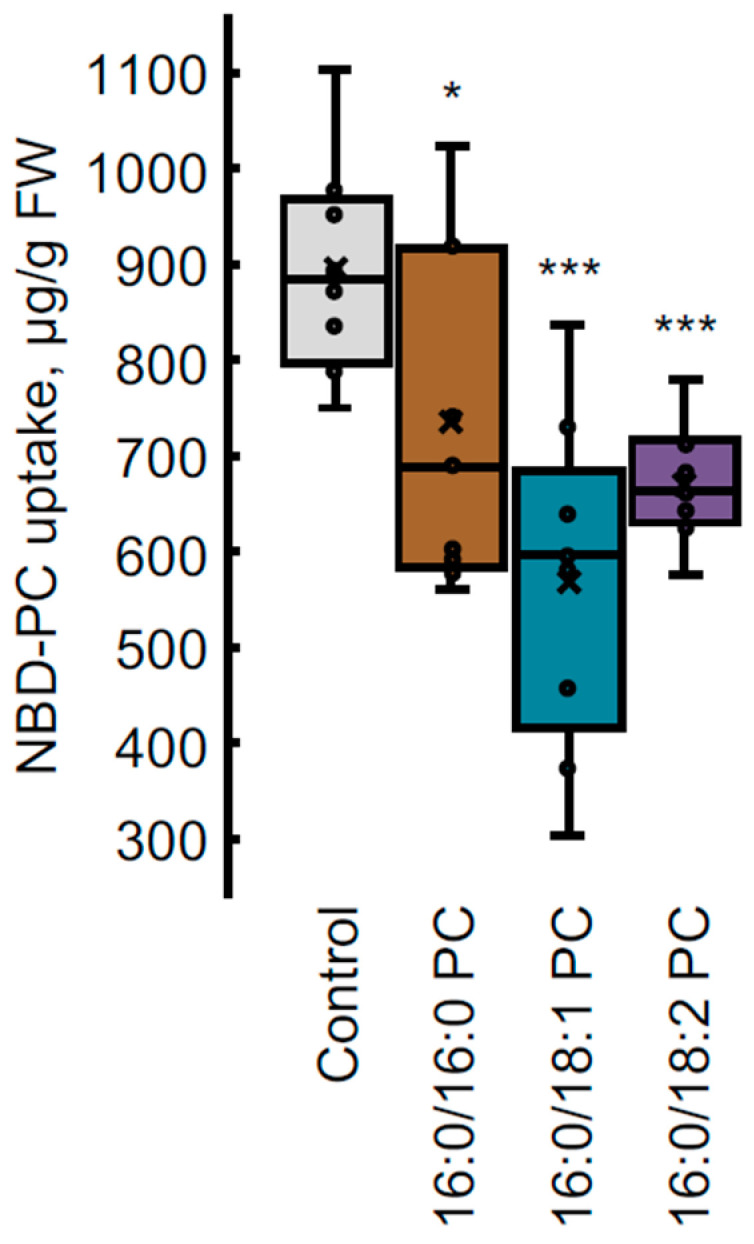
NBD-PC uptake in seven-day-old *A. thaliana* seedling roots grown on half-strength Murashige–Skoog medium (control) supplied with 20 μM 16:0/16:0 PC, 16:0/18:1 PC, or 16:0/18:2 PC. After 1 h incubation in 40 μM NBD-PC in TRIS/MES buffer (2 mM/4 mM, respectively, pH 6.0), NBD-PC was extracted from roots and analyzed with HPLC-FLD. Data represented as a box from 1st quartile to 3rd with marked median (horizontal line in the box) and mean (× symbol); *n* = 9 from three biological replicates. *** *p* ≤ 0.001, * *p* ≤ 0.05, significantly different with respect to control based on Student’s *t*-test.

For more precise quantification of NBD-PC uptake, lipids were extracted from roots after NBD-PC treatment and analyzed with HPLC-FLD analysis. As an example, control plants and seedlings grown with 16:0/16:0 PC, 16:0/18:1, and 16:0/18:2 PC were chosen. According to the results of NBD-PC quantification depicted in Figure 5, plants accumulated fluorescent lipids in the amount of 0.5–1 mg/g FW. A correlation was observed between patterns of NBD-PC internalization obtained with confocal microscopy and NBD-PC uptake quantification with HPLC-FLD analyses, which proved the objectivity of data obtained with confocal microscopy. Quantification of internalized NBD-PC in Arabidopsis roots confirmed that seedlings grown on the medium supplemented with PC 16:0/16:0, PC 16:0/18:1 or PC 16:0/18:2 accumulated significantly lower amounts of the probe. For instance, PC 16:0/16:0 in the medium resulted in 17.8% less uptake of NBD-PC, followed by −25% on PC 16:0/18:2, and 36.3% less NBD-PC uptake on PC 16:0/18:1.

### 2.3. The Structure of Exogenous PC Molecular Species Affects the A. thaliana Lipidome

The methodological basis of the conducted targeted lipidomic studies was liquid chromatography with triple quadrupole tandem mass spectrometry (LC-QqQ-MS/MS) performed in the multiple reaction monitoring (MRM) mode. This method was chosen due to its high sensitivity for the detection of minor molecular species and selectivity towards iso-elemental species, as well as its higher quantitative accuracy. To make a table of the transitions from selected precursor ions to fragment ions in the MRM mode, a preliminary scan of the PC and PE mixtures in positive ion mode using monitoring of the product ion of *m*/*z* 184 and neutral loss scan of fragment *m*/*z* 141 was performed to determine all PC and PE molecular species, respectively. A full table of transitions has been compiled for 120 molecular species of PC and 186 PE to determine all possible molecular species corresponding to the resulting mass list (Table S1). Using this method, 30 molecular species for PC and 45 for PE were identified and quantified (Table S2).

**Figure 6:**
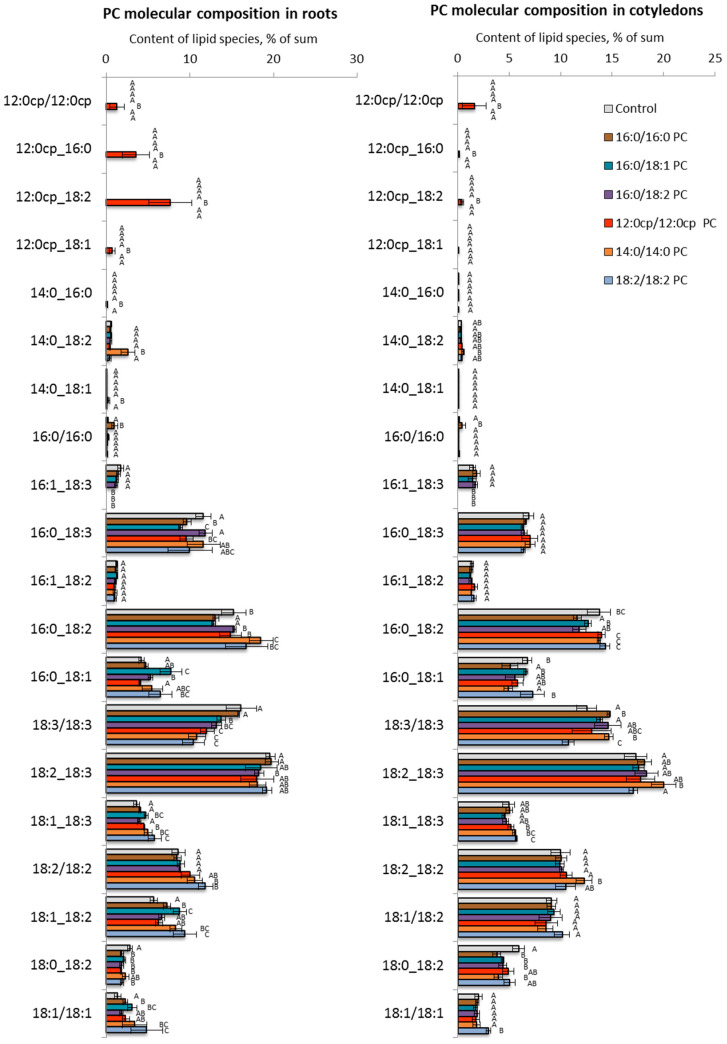
The effect of PC molecular species on the endogenous PC molecular profile of *A. thaliana*. The experiments were performed in biological triplicate, and error bars indicate standard deviations. Different letters above the bars indicate statistically significant differences at *p*-value < 0.05 (*t*-test).

Plants exposed to media with 12:0cp/12:0cp PC, 14:0/14:0 PC, or 16:0/16:0 PC, i.e., with molecular species that are not synthesized in *Arabidopsis* (12:0cp/12:0cp PC) or synthesized in trace amounts (14:0/14:0 PC, 16:0/16:0 PC), could be used to judge the process of uptake of exogenous lipids. In our study, only internalization of 12:0cp/12:0cp PC (up to 1.5% of sum) and 16:0/16:0 PC (up to 1%) was reported (Figure 6). 14:0/14:0 PC, once in the cell, rapidly undergoes conversion to form the 14:0/16:0 PC (0.1%), 14:0/18:1 PC (0.3%), 14:0/18:2 PC (3%), and 14:0/18:2 PE (1.6%) species. Similar chimeric structures, only in greater amounts, were reported in the experiment with 12:0cp/12:0cp PC, including 12:0cp/16:0 PC (3.5%), 12:0cp/18:1 PC (0.7%), 12:0cp/18:2 PC (8%), and 14:0/18:2 PE (0.6%). The uptake of other molecular species of PC that are natural for *Arabidopsis* (16:0/18:1 PC, 16:0/18:2 PC, and 18:2/18:2 PC) cannot be assessed. Nevertheless, the absence of significant changes in the relative content of the corresponding molecular species indicates their intensive conversion and/or intensification of processes aimed at avoiding disturbing membrane lipid homeostasis.

**Figure 7:**
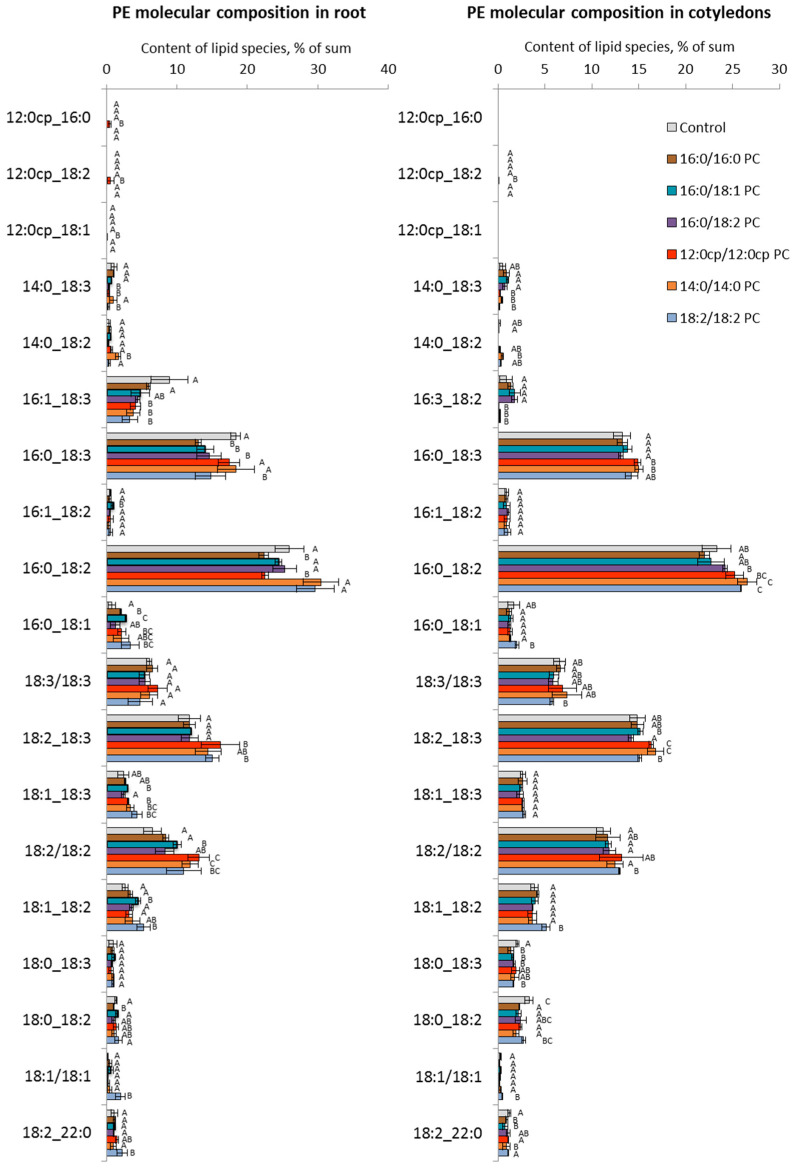
The effect of PC molecular species on the endogenous PE molecular profile of *A. thaliana*. The experiments were performed in biological triplicate, and error bars indicate standard deviations. Different letters above the bars indicate statistically significant differences at *p*-value < 0.05 (*t*-test).

To further detail the changes in GPL profiles, we analyzed molecular species of PC and PE separately for roots growing on the surface of the nutrient medium, with lipids and aerial parts (cotyledons) being minimally in contact with the medium. We also identified several typical changes that develop in response to exogenous lipids. The main trend in the remodeling of PCs (Figure 6) and PEs (Figure 7) in roots when introducing exogenous 16:0/16:0 PC and 16:0/18:1 PC was a decrease in the relative content of 16/18 PC and 16/18 PE that was compensated for by the increased level of 18/18 PC and 18/18 PE, respectively. Another response to the exposure to these exogenous lipids was an increase in the level of molecular species containing C18:1, including 18:1/18:1 PC, 18:1/18:2 PC, 18:1/18:2 PE. The changes in the cotyledons were not significant.

The lipid profile of plants grown on medium with 16:0/18:2 PC and 12:0cp/12:0cp changed little; however, decreased levels of 18:3/18:3 PC, 16:0/18:3 PE, or 16:1/18:3 PE were recorded. Changes in cotyledons were also insignificant.

A more specific response was detected when 14:0/14:0 PC and 18:2/18:2 PC were supplied. For example, in roots, an increase in the relative content of molecular species acylated by C18:1 acid when grown on a medium with 18:2/18:2 PC was shown. Under the influence of 14:0/14:0 PC, cotyledons showed an increase in the relative content of 18:3/18:3 PC, 18:2/18:3 PC, and 18:2/18:3 PE. In response to 18:2/18:2 PC, there was an increase in the level of molecular species with C18:1, e.g., 18:1/18:1 PC, and 18:1/18:1 PE, 18:1/18:2 PE. It could be observed that, in contrast to roots, changes in PC and PE in cotyledons appeared to be more consistent.

**Figure 8:**
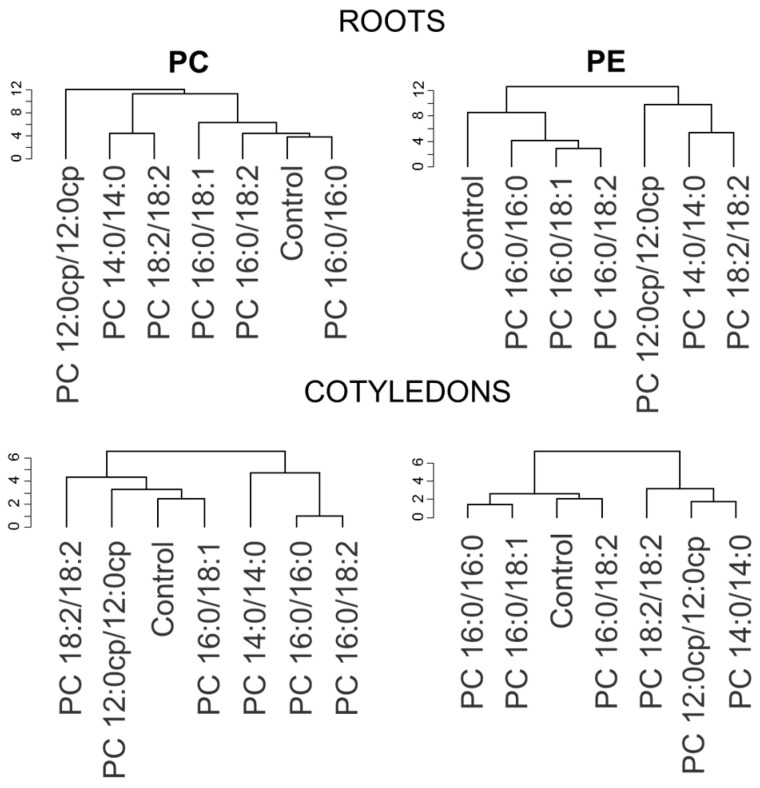
Dendrograms of hierarchical clustering of lipidomic data.

Analysis of changes in the lipidome of plants grown on media with exogenous lipids using hierarchical clustering showed that 2–3 groups could be distinguished according to changes in individual molecular species within PC and PE (Figure 8). One group included the lipidomes of control plants, as well as plants grown with 16:0/16:0 PC and 16/18 PC. The other group consisted of plants grown with 14:0/14:0 PC and 18:2/18:2 PC. The third group is represented by lipidomes of plants grown on medium with synthetic lipid 12:0cp/12:0cp PC.

**Figure 9:**
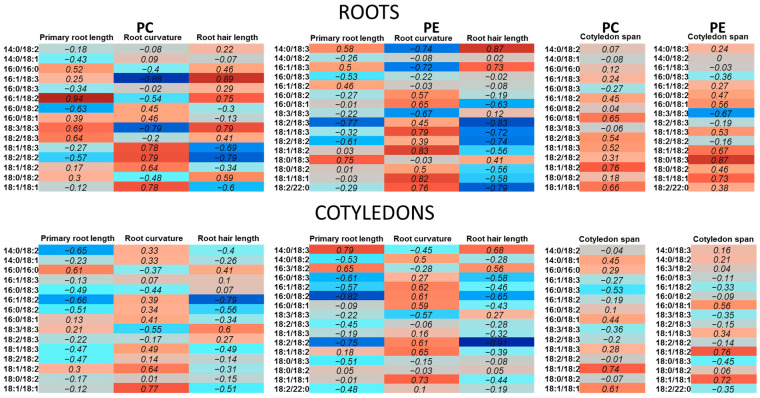
Heat map showing Pearson’s correlation coefficients for lipidomic and morphometric parameters. Colors correspond to correlations values in the cells, blue = negative, red = positive.

To understand the relationship between lipidomic data and plant morphological changes, heat maps visualizing Pearson’s correlation coefficients for lipidomic and morphometric parameters were constructed (Figure 9). On the heat maps, the positive values marked red correspond to positive correlations between morphometric parameters and contents of lipid species and vice versa. This method helped to uncover several trends, which will be described in the Section 3.

## 3. Discussion

It has been recently shown that fungi and plants are not only capable of synthesizing the entire spectrum of lipids de novo but also possess a well-developed system that allows them to assimilate exogenous lipids [51,52,53,54,55]. This finding raised new questions concerning the unique role of lipid molecules, the mechanisms of their uptake, and metabolic conversion, as well as their participation in the interaction of organisms with the environment. However, the dependence of the ability to absorb and metabolize lipids on their structure has not yet been characterized in detail. In the present work, the in vivo effects of exogenous PC molecular species with different structures and physical properties on the model plant *Arabidopsis thaliana* were studied using macromorphological, micromorphological, and lipidomic approaches. Our goal was to provide insights into the uptake and metabolic conversion of exogenous GPLs as a function of their molecular chemistry. In order to approach an understanding of the natural processes that occur when plant roots come into contact with soil humus containing plant residues, soil bacteria, and fungi, the saturated molecular species of PCs (14:0/14:0 PC, 16:0/16:0 PC) typical of bacteria, and the unsaturated molecular species of PCs (16:0/18:2 PC, 18:2/18:2 PC) typical of fungi and plants, were selected for study. For comparison, the effect on morphology and lipid metabolism of an artificially synthesized (1,2-di-(3-(3-hexylcyclopentyl)-propanoate)-*sn*-glycero-3-phosphatidylcholine with cyclopentane (cp) rings incorporated into C12:0 chains (12:0cp/12:0cp PC), which is close in structure to archaeal lipids and whose physical properties have been studied previously [56], was also investigated. The 16:0/18:1 mixed-chain combination is fairly universal for eukaryotic GPLs, particularly PCs, and has been found in fungi [57] and plants [58,59,60], as well as within the soil lipidome [38].

The composition of membrane lipids can be highly variable under changing environmental conditions. It is one of the most evolutionarily ancient adaptive responses at the level of low-molecular-weight metabolites [42,61,62]. Changes in lipid composition can initiate the activation of cellular stress responses and affect key metabolic pathways essential for growth [38]. Membrane lipids can be remodeled to meet new requirements for the physical properties of membranes and to conserve energy and optimize the use of nutrients such as nitrogen and phosphorus [16,63,64,65]. They can also be used as an endogenous source of carbon and energy [42]. However, in some cases, the maintenance of lipid homeostasis may become the main membrane strategy in response to changes in the environment [65]. This response is characteristic, for example, of the development of ER stress, which provides protein quality control so that only properly folded proteins leave the ER [66]. It is based on changes in the expression of a whole family of PC and PE metabolism genes. Their coordinated changes maintain a constant ratio of PC and PE, as well as the composition of their fatty acids [66]. However, homeostasis at the class and fatty acid levels does not indicate maintenance of equilibrium at the molecular species level. Therefore, true lipid metabolism homeostasis can only be judged based on the results of lipidome analysis. 

In our study, supplementation with exogenous PCs resulted in only minor changes in growth and morphology. This is despite the fact that, as shown in our work and others [30], exogenous lipids are easily incorporated into plants in a tissue-specific manner (in cells of the root tips and hairs). It was confirmed that P4-type ATPase flippase ALA10 with the ALIS1 complex facilitates internalization of externally applied phospholipids, including PC, lyso-PC, PE, and PS [30]. According to our results, plants uptake completely different PC molecular species from typical-to-them unsaturated ones such as 16:0/18:2 PC, 18:2/18:2 PC, to untypical, entirely saturated ones including 14:0/14:0 PC, 16:0/16:0 PC, and exotic 12:0cp/12:0cp PC with cyclopentane rings. Saturated “bacterial” lipids and typical plant unsaturated lipids can differently affect *Arabidopsis* membranes. 16:0/16:0 PC and 16:0/18:1 PC appear to be incorporated into membranes both intact and after editing, followed by changes in membrane properties. In particular, they made membranes less permeable to the lipid NBD-containing fluorescent probe. Indeed, straight-chain molecules such as the 16:0/16:0 PC pack together tightly and decrease membrane fluidity and permeability [42]. However, the observed cell response may be related not so much to the physical properties of the exogenous 16:0/16:0 PC and 16:0/18:1 PC as to its biology. Apparently, long-chain lipids are easily recognized by enzymes of lipid catabolism and quickly metabolized to meet the needs of cells. At the morphological level, these reactions cause only minor changes in the length of roots and root hairs. 

As for 14:0/14:0 PC and 18:2/18:2 PC, their penetration into the membrane is accompanied by apparently rapid disassembly of the molecules. We found no evidence for the long-term existence of these exogenous molecules in membranes in the intact state, but the accumulation of 14:0/18:2 PC and 14:0/18:2 PE, as well as 16:0/18:2 PC and 16:0/18:2 PE, upon exposure to 14:0/14:0 PC and molecular species with 18:1 upon exposure to 18:2/18:2 PC, may indicate their conversion. These C18:1-, C18:2-contained molecules, as shown by NBD experiments, can sufficiently keep a high level of membrane permeability. Indeed, bent or kinked chains with cis double bonds create bulky or porous layers that increase membrane fluidity and permeability [42]. The uptake of exogenous lipids undoubtedly leads to changes in the plasma membrane. Regarding C14:0, it is known that, in plant cells, there is a mechanism to avoid bottleneck 14/16 PC and 14/18 PC accumulation in the membrane [24,59]. PCAT, LPCAT, and diacylglycerol acyltransferases (DGAT), as well as FATB, are the main participants in the detoxification round which is terminated by the flux of C14:0 to TAG production [59]. Plants grown on artificially synthesized 12:0cp/12:0cp PC showed the most striking response to exposure. We have revealed that lipids of this type, often found in bacteria, can cause significant changes both at the level of the whole lipidome and cell membranes. Apparently, such GPL-esterified fatty acids are poorly recognized by plant hydrolyzing enzymes and are slow to metabolize. This may result in their incorporation into the membrane, which causes an increase in membrane permeability due to their pore-forming ability [56]. This process is quite toxic to the cell, which is manifested at the morphological level.

A detailed targeted analysis of PC and PE molecular species, including minor ones, allowed us to characterize not only the incorporation and conversion of exogenous lipids but the response reactions of GPL metabolism aimed at maintaining lipid homeostasis or, on the contrary, compensatory adaptive changes. According to the results observed, the local changes in the membrane seem to be short-lived because the incorporated lipids are rapidly metabolized by a complex of enzymes. In particular, phospholipases, PCATs, and LPCATs are strong candidates to participate in such metabolic conversion [67,68]. It appears that homeostasis systems do not allow a complete unbalancing of the lipid profile. This is supported by feeding experiments in which different organisms can metabolize a range of fatty acids without damage [69,70,71,72]. Nevertheless, changes in the composition of the lipid profile can be observed.

Hierarchical clustering of lipidomes of plants grown on media with exogenous lipids revealed several groups with common trends in the variation of individual molecular species of PC and PE within the lipidome (Figure 8). The major trends in the lipidome response in these groups are the changes in the ratios of 16/18 and 18/18 molecular species and the level of species with C18:1 and C18:2. It is significant whether the balance shifts in the direction of an increase in the proportion of saturated or unsaturated fatty acids. Saturated fatty acids are precursors for sphingolipid, surface wax, and cutin biosynthesis and are involved in protein acylation. Mutant plants with a 50% reduction in saturated fatty acids demonstrate a decrease in growth rate and deformed development of seeds [20]. However, as has already been demonstrated in animal models, some saturated acids such as C16:0 cause cellular damage, including apoptosis, oxidative stress, ER stress, and mitochondrial dysfunction [73]. Interestingly, a mono-unsaturated fatty acid (C18:1) was able to alleviate saturated fatty acids-induced toxicity both in vitro and in vivo. In our study, we also observed an increase in oleic acid-containing molecular species (18:1/18:1 PC, 18:1/18:2 PC, 18:1/18:3 PC) after the incubation of plants with 16:0/16:0 PC, 16:0/18:1 PC, and 18:2/18:2 PC. According to results presented in the heatmap of correlation coefficients (Figure 9), 18:1/18:1 PC, 18:1/18:1 PE, and some other C18:1 molecular species are positively correlated with root curvature and the size of cotyledons. Plants with this lipidomic trend showed the most developed roots and/or enhanced growth of the aerial parts.

Unsaturated fatty acids play a special role as precursors of signaling molecules. For example, their reduction leads to a weakening of the defense reactions that develop during pathogenesis [74]. Calculation of the correlation coefficients showed that the accumulation of C18:3-containing molecular species such as 18:3/18:3 PC, 18:2_18:3 PC, 16:1_18:3 PC, 16:1_18:3 PE, and 14:0_18:3 PE, as well as 16:1_18:2 PC, is positively connected with the highest length of primary roots and root hairs. At the same time, these molecular species are negatively correlated with the root curvature index (Figure 9). We observed the highest relative content of these molecular species in roots of actively growing control plants, as well as in roots of plants grown on 16:0/16:0 medium. This finding is consistent with our past observations, where an accumulation of C18:3-containing phospholipids (18:3/18:3 PC, 18:3/18:3 PE, 18:2/18:3 PC, 18:2/18:3 PE) was observed in the growing edge of the filamentous fungal colony enriched in vegetative, actively growing hyphae [75]. Such an analogy suggests that the synthesis of 18:3-containing molecular species of PCs and PEs is associated with the monopolar growth of different cells and organs and is a kind of universal reaction of taxonomically distant organisms.

Thus, the observed responses to the exposure to exogenous lipids may be due not only to the physical properties of exogenous lipid molecules but also to their cell functions. This finding has raised new questions concerning the unique role of the structure of lipid molecules, the mechanisms of their uptake and metabolic conversion, and their involvement in the interaction of organisms with the environment.

## 4. Materials and Methods

### 4.1. Plant Material and Growth Conditions

Lipid uptake experiments on plants grown on various PC molecular species were performed using 7-day-old seedlings of *Arabidopsis thaliana* (L.) Heynh ecotype Col-0. At this stage, morphological changes in the primary root, which reacts most actively to the effects of exogenous lipids, were clearly visible, and the observations did not interfere with lateral roots. Seeds were thoroughly surface-sterilized (1 min vortexing in 70% ethanol followed by 7.5 min vortexing in 42% bleach containing 2% Triton X-100 *v*/*v*, then 5× rinse and vortexing in sterile water) and then pipetted onto the surface of solidified half-strength Murashige−Skoog nutrient medium (MS/2, Duchefa M0222.0010, the Netherlands) containing 1% (mass/vol) sucrose (“Ecros”, St. Petersburg, Russia) and 0.35% (mass/vol) phytagel (“Sigma-Aldrich”, St. Louis, MO, USA) in 100 mm square Petri dishes (“Sarstedt”, Nürmbrecht, Germany). PC lipids, including artificially synthesized (1,2-di-(3-(3-hexylcyclopentyl)-propanoate)-*sn*-glycero-3-phosphatidylcholine with cyclopentane (cp) rings incorporated into C12:0 chains (12:0cp/12:0cp PC) [56], as well as 14:0/14:0 PC, 16:0/16:0 PC, 16:0/18:1 PC, 16:0/18:2 PC, 18:2/18:2 PC (Avanti Polar Lipids, Alabaster, Montgomery, AL, USA), were added to the medium, simultaneously with pouring of autoclaved medium in Petri dishes from 2 mg/mL stocks in methanol to a final concentration of 20 μM in the medium. After stratification (48 h at 4 °C), plates were placed vertically for 7 days in the MLR-351 climate chamber (Sanyo, Tokyo, Japan) at 20 °C with a 12 h/12 h light/dark cycle and at 100 μmol⋅m^−2^⋅s^−1^ illuminance provided using FL40SS-W/37 fluorescent lamps (Panasonic, Tokyo, Japan).

### 4.2. Seedling Measurements

Digital images of plants were taken with 2× lens of Xiaomi 11 Lite, then geometry-corrected in Adobe Camera RAW. Images were processed in ImageJ (version 1.52p) or FIJI (v. 2.0.0-rc-69/1.52p) for measurements: root length was measured by tracing the primary root with the “segmented line” tool from the root collar, along the root, to the root tip. The same approach but using a “straight line” tool was applied to measure hypocotyl width-span. Root curvature (dimensionless units) was calculated as the root length divided by the length of the straight line drawn between the root collar and the root tip. At least 40 seedlings were examined for each experimental condition: without supplementation (control) or with supplementation of PC lipids (12:0cp/12:0cp PC, 14:0/14:0 PC, 16:0/16:0 PC, 16:0/18:1 PC, 16:0/18:2 PC, 18:2/18:2 PC). 

### 4.3. Lipid Uptake Visualization

To visualize lipid uptake, plants were bathed for 30 min in 40 μM solution of 18:1-06:0 NBD PC (Avanti Polar Lipids, Alabaster, Montgomery, AL, USA) in TRIS/MES buffer (0.4 mM/0.2 mM, pH 6.0) prepared from 10 mM stock in DMSO, then rinsed three times for 5 min in the clean buffer; roots were cut from aerial part of the seedlings, and then each of the three roots was mounted per objective slide for microscopy. Confocal microscopy experiments were carried out using a Leica TCS SP5 MP inverted confocal laser scanning microscope (Leica Microsystems, Wetzlar, Germany), equipped with a 40× objective lens (NA 1.3, oil immersion), or using Carl Zeiss LSM 780 microscope (Carl Zeiss AG, Oberkochen, Germany) under 20× objective lens (NA 0.8). NBD-PC fluorescence was induced with a 488 nm argon laser, and emission was recorded in the range of 510–560 nm. Z-stacks of 10 to 18 optical sections (387.88 × 387.88 μm or 258.59 × 258.59 μm) were recorded at 2.5 μm intervals. Figures show maximal intensity projections of a substack from the central part of the acquired z-stack. Source data were inspected in the manufacturer’s software (LAS AF Suite 2.6 for Leica, ZEN 2012 for Zeiss), and substacks and maximal intensity projections were prepared in FIJI software (v. 2.0.0-rc-69/1.52p).

### 4.4. NBD-PC Uptake Measurement by HPLC-FLD Analysis

HPLC-FLD analysis was performed on the LicArt62 HPLC system (Labconcept, St. Petersburg, Russia), equipped with a fluorescence detector. From each Petri dish, three groups of five 7-day-old Arabidopsis seedlings per group were isolated, then incubated in 300 μL of 40 μM NBD-PC solution in TRIS/MES buffer (0.2 mM/0.4 mM, pH 6.0) for 60 min at room temperature, then rinsed twice in the same clean buffer for 10 min. Then the aerial parts were cut from the roots, the roots were surface-dried with filter paper and stored in microcentrifuge tubes at −80 °C upon extraction. For extraction, 50 μL of methanol was added to each sample, which was ground thoroughly with a pellet pestle and centrifuged for 1 min at 10,000 rpm, and the supernatant was transferred to chromatography vial with 200 μL conical insert. For chromatographic separation, the Agilent Eclipse XDB-C18 column (150 × 2.1 mm, 3.5 μm) was used. The mobile phase consisted of (A) water/methanol (1:1 *v*/*v*), and (B) 2-propanol/formic acid (99.9:0.1 *v*/*v*). Analyses were performed at 45 °C and a flow rate of 0.3 mL min^−1^ in the gradient elution mode, and the percentage of B was programmed as follows: 45% (0 min)–90% (10 min) 90% (15 min)–45% (20 min). The volume of the injected sample was 5 μL. Detection of NBD-PC was carried out using the fluorescence detector, the excitation wavelength was 464 nm, and emission was registered at 531 nm. To identify NBD-PC in samples, we compared retention time (Rt) with the commercially available authentic standard NBD-PC (810132P-10 mg, Avanti Polar Lipids, Alabaster, Montgomery, USA). The content of NBD-PC in a sample was calculated based on the calibration curve with concentrations of 0.5, 1.0, 2.0, and 4.0 μM and then normalized to the fresh weight of roots in the sample (Figure S1).

### 4.5. Lipid Extraction, Separation and Analysis

#### 4.5.1. Lipid Extraction

Seven-day-old seedlings of *A. thaliana* (approximately 80 seedlings per Petri dish resulting in one replicate) were removed from the nutrient medium and cut into two parts–root and aerial part of seedling consisting of cotyledon and hypocotyl 2 mm above root collar, referred to herein as “cotyledon”. Both parts were extracted for lipids separately. The plant material was homogenized in a mortar with 3 mL of chloroform: methanol 1:2 (*v*/*v*) mixture and samples were incubated at 4 °C overnight in glass tubes [76]. Next, the samples were centrifuged to remove cell debris; 1 mL chloroform and 1.5 mL 2.5% NaCl were added for phase separation. The bottom chloroform fraction containing total lipids was collected and samples were dried in IKA rotary evaporator (Ika, Staufen, Germany).

#### 4.5.2. Separation of Lipid Classes by TLC

PC and PE were separated using two-dimensional thin-layer chromatography (TLC) on silica gel 60 10 × 10 cm plates (Merck, Darmstadt, Germany) in a solvent system chloroform:methanol:water (65:25:4) in the first direction and chloroform:acetone:methanol:acetic acid:water (50:20:10:10:5) in the second direction [77]. After temporary visualization in iodine vapors, PC and PE spots were scrapped from TLC plates and eluted with chloroform:methanol (1:2) at 4 °C overnight, then evaporated and redissolved in 40 µL HPLC-grade methanol. Quality control samples for roots and leaves were prepared by combining 5 μL of each sample extract.

#### 4.5.3. Lipid Profiling

Lipid profiles of PC and PE were analyzed by LC-MS/MS using LCMS-8030 triple quadrupole instrument (Shimadzu, Kyoto, Japan) with a Nexera UHPLC system (Shimadzu, Kyoto, Japan). The samples were loaded onto a Kinetex C18 LC column (2.6 μm, 2.1 × 150 mm, 100 Å, Phenomenex, Torrance, CA, USA) using an SIL-30AC autosampler held at 4 °C. 

The flow rate was 0.3 mL/min, and the column oven temperature was set at 50 °C. Mobile phases were as follows: acetonitrile/water 1:1 (*v*/*v*) as solvent A and 2-propanol/acetonitrile/water 85:10:5 (*v*/*v*/*v*) as solvent B, both containing 5 mM ammonium formate and 0.1% (*v*/*v*) formic acid [78]. The gradient program was as follows: 1 min, 45% B; 11 min, 90% B; 11.1 min, 100% B; 15 min, 100% B; 15.1 min, 45% B; 16 min, 45% B (stop time). The following parameters were used for ionization: nebulizing gas flow 3.0 L/min, drying gas flow 15.0 L/min, DL temperature 250 °C, heat block temperature 400 °C; ESI interface voltage, 4500 V (+), 3500 V (−).

Two-stage MS/MS methodology was used for phospholipid profiling [79]. The precursor ion scan for PC and neutral loss scan for PE were used as the first untargeted stage of lipid profiling to screen for functional groups associated with particular lipid classes. PC profiling was performed in positive ion mode using monitoring of the product ion of *m*/*z* 184. The parameters were set as follows: MS1 mass range, 630–1050 *m*/*z*, event time, 100 ms; collision energy, −34 V; Q1 resolution, high; Q3 resolution, unit. The neutral loss scan of fragment *m*/*z* 141 in positive ion mode was used to determine PE molecular species. The parameters were set as follows: MS1 mass range, 630–1050 *m*/*z*, event time, 300 ms; collision energy, −20 V; Q1 resolution, high; Q3 resolution, unit.

Based on the data of this untargeted approach, a panel of phospholipid multiple reaction monitoring (MRM) transitions was prepared using detected molecular masses of phospholipids and the theoretical values of fatty acid-related fragmentation. The full list of MRMs and MS parameters are shown in the Supplementary Table S1. MRM parameters: dwell time, 5 ms; Q1 and Q3 resolution, unit.

Blank control and three quality control (QC) samples were regularly interspersed to ensure the quality of the batch. Peak areas of each molecular species were normalized to the sum of peak areas of all molecular species within the same lipid class. An equimolar mixture of 13:0/13:0 PC, 15:0/15:0 PE, and 19:0/19:0 PC molecular species (Avanti Polar Lipids, Alabaster, Montgomery, AL, USA) indicated that the signal intensities reflect the relative abundance of the phospholipid molecular species under the conditions used.

Data were processed in LabSolutions Postrun Analysis software 5.98SP1 (Shimadzu) and Skyline [80]. 

### 4.6. Statistical Analysis

All experiments were performed in at least three biological replicates. Independent plants taken from different Petri dishes for micro- and macromorphological analyses, or several plants grown in the same Petri dish (for HPLC and HPLC-MS analyses), were considered as one biological sample. The number of independently inspected seedlings in morphological studies varied depending on the method used, from 7–9 for confocal microscopy of NBD-PC fluorescence to 30–120 for macromorphology analysis. For one biological sample, 5 and 70–80 seedlings were selected for HPLC analysis of NBD-PC uptake and HPLC-MS analysis of lipid profile, respectively. Three independent experiments were conducted for one year. The results of all three experiments for micro- and macromorphology data, and the most representative experiment for HPLC and HPLC-MS analyses, are presented in this paper. Statistical analysis of the data was performed in Microsoft Excel program; significance was evaluated using Student’s *t*-test. Data presented in histograms are mean values with standard errors. For statistical calculations and visualization, R 4.3.1 “Beagle Scouts” was used [81]. Hierarchical clustering was performed using Euclidean distances and Ward’s method. Morphometric data were autoscaled to prevent the influence of scale differences between measures of different natures.

## Data Availability

The data presented in this study are available on request from the corresponding author. The data are not publicly available due to privacy restrictions.

## Supplementary Materials

The supporting information can be downloaded at: https://www.mdpi.com/article/10.3390/ijms25010089/s1.
