# Supplementary material for: Uptake and Metabolic Conversion of Exogenous Phosphatidylcholines Depending on Their Acyl Chain Structure in Arabidopsis thaliana
Source: Int J Mol Sci. 2023 Dec 20;25(1):89. doi: 10.3390/ijms25010089 (PMC10778594; doi:10.3390/ijms25010089)
Supplement: Supplementary file 1 [file ijms-25-00089-s001.zip › Figure S1 NBD-PC HPLC calibration curve.pdf]

International Journal of
Molecular Sciences

MDPI

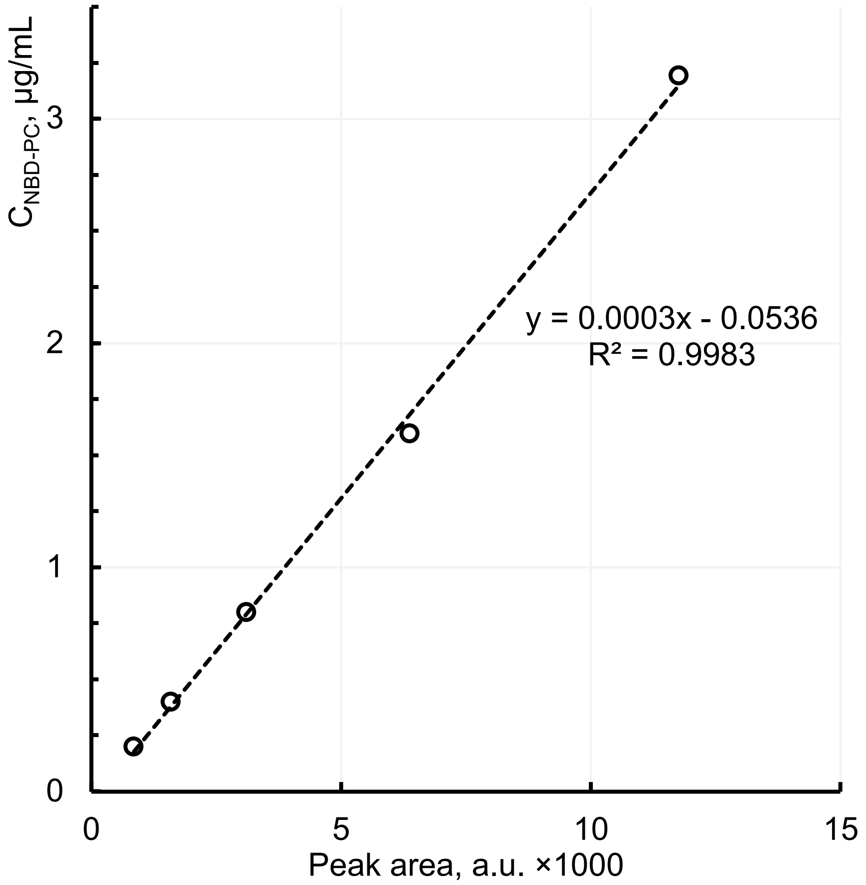

Figure S1. Calibration curve for NBD-PC measurement using HPLC-FID. Peak area from chromatogram was derived using LicArt WSV Software 00.01.01.08 (Labconcept, Russia). NBD-PC was diluted from 10 mM stock solution in DMSO to a final concentrations of 0.25, 0.5, 1.0, 2.0 and 4.0 μM in methanol (0.199, 0.399, 0.798, 1.596 and 3.192 μg/mL, respectively). Molar concentration was converted to mass content given that molar mass of 18:1-06:0 NBD PC equals to 797.434 g/Mol. Linear regression equation and corresponding coefficient of determination are displayed below the trend line.
